# Higher quality nutrition care process documentation predicts nutrition diagnosis improvement in the Academy of Nutrition and Dietetics breastfeeding registry study

**DOI:** 10.3389/fnut.2025.1632931

**Published:** 2025-11-17

**Authors:** Allison Gaubert, Julie M. Long, Lindsay Woodcock, Lauri Wright, Casey Colin, Hanadi Hamadi, Constantina Papoutsakis

**Affiliations:** 1 Department of Allied Health Sciences, College of Science and Technology, Nicholls State University, Thibodaux, LA, United States; 2 Data Science Center, Research, International, and Scientific Affairs (RISA), Academy of Nutrition and Dietetics, Chicago, IL, United States; 3 College of Public Health, University of South Florida, Tampa, FL, United States; 4 Department of Nutrition and Dietetics, Brooks College of Health, University of North Florida, Jacksonville, FL, United States; 5 Department of Public Health, University of North Florida, Jacksonville, FL, United States; 6 Center for Aging Research, Brooks College of Health, University of North Florida, Jacksonville, FL, United States

**Keywords:** lactation, breastfeeding, registered dietitian nutritionist, nutrition care process, nutrition care process terminology, informatics, medical nutrition therapy, public health nutrition

## Abstract

**Introduction:**

Registered dietitian nutritionists (RDNs) provide medical nutrition therapy (MNT) to improve public health outcomes, yet RDNs impact on breastfeeding outcomes remains underexplored. The Breastfeeding Registry Study addresses this gap by examining MNT provided to breastfeeding infants. This study describes Nutrition Care Process (NCP) documentation patterns, evaluates documentation quality, and reports nutrition diagnosis improvement, goal attainment, and outcomes predictors.

**Methods:**

This prospective, observational study included documentation from breastfeeding infants (*n* = 92) from July 2020 to June 2024 using the Academy of Nutrition and Dietetics Health Informatics Infrastructure. The primary outcome was breastfeeding duration. Frequencies of documented NCP terminology, impactful care plans, and nutrition diagnosis improvement were assessed. Documentation quality was evaluated using the NCP Quality Evaluation and Standardization Tool (NCP-QUEST). Mixed-effects logistic regression was used to identify predictors of improved outcomes.

**Results:**

Duration of any breastfeeding averaged 34.2 ± 7.5 (mean ± SD) days (*n* = 10), although documentation of this indicator was poor. The most frequent etiology was breastfeeding difficulty (18%). Common intervention categories were Food and/or Nutrient Delivery (46%) and Coordination of Nutrition Care (43%). At reassessment, 68% of diagnoses improved, with the highest rates for breastfeeding difficulty (55%), predicted breastfeeding difficulty (83%), inadequate vitamin D intake (83%), and underweight (83%). NCP-QUEST score (OR = 1.58, 95% CI [1.02, 2.45] *p* = 0.042) and frequency of registered dietitian visits (OR = 1.77, 95% CI [0.34, 0.9.33] *p* = 0.049) predicted diagnosis improvement.

**Discussion:**

Higher-quality documentation and more RDN visits were associated with improvements in breastfeeding infants' nutrition diagnoses. This is the first known study to describe comprehensive care plans delivered by RDNs that improved prevalent lactation-related nutrition problems and to propose standards for documenting breastfeeding care data in alignment with global breastfeeding standards.

## 1 Introduction

National and global health authorities recommend breastfeeding as the cornerstone of early life nutrition (1–4). The World Health Organization (WHO) and the United Nations Children's Fund (UNICEF) recommend exclusive breastfeeding for the first six months of life, followed by continued breastfeeding alongside complementary foods for up to 2 years and beyond (5). Registered dietitian nutritionists (RDNs) provide medical nutrition therapy (MNT) across various practice settings to support breastfeeding (6–10). Their education and training prepare them to address common breastfeeding challenges (7, 9–11), including latching difficulties, sore nipples, insufficient milk supply, and breast engorgement, which frequently disrupt exclusive breastfeeding and contribute to public health burden (5, 11–13). RDN care may include individualized education and breastfeeding plans, and coordination of lactation strategies such as donor human milk use (10), which can reduce healthcare costs and complications like necrotizing enterocolitis (14). Despite this critical role, few studies have evaluated the impact of RDN-delivered MNT on breastfeeding outcomes aligned with WHO/UNICEF breastfeeding standards (5), leaving their contributions in this area of public health underexplored.

RDNs use the Nutrition Care Process (NCP) Model (15), which consists of the nutrition assessment/reassessment, diagnosis, intervention, and monitoring and evaluation steps. As nutrition care is documented using the standardized NCP Terminology (NCPT) (15), five NCP chain links may be formed, making connections between the evidence, diagnosis, etiology, goals, and outcomes, which reflect critical thinking across the continuum of care (16). The Academy of Nutrition and Dietetics (Academy) Health Informatics Infrastructure (ANDHII) (17) is a secure, web-based platform for documenting nutrition care and conducting nutrition and dietetics registry studies (18). Registry studies explore practice patterns, outcomes, and quality of care, supporting the Academy's efforts in performing practice-based NCP research (18). Emerging evidence indicates that higher quality NCP documentation predicts greater nutrition diagnosis improvement in adult populations. This suggests a connection between high-quality documentation and improved outcomes (19, 20). While prior analyses of NCP documentation have assessed outcomes including diagnosis resolution, goal progress, and health indicators across diverse practice areas, none have included lactation nutrition (19–28).

Quantifying RDN impact is critical, and the effect of MNT on breastfeeding-related outcomes remains unclear. To address this gap, the Breastfeeding Registry (BFR) Study aimed to: (1) identify the most frequently documented NCPT; (2) assess NCP documentation quality; and (3) report on nutrition diagnosis improvement and resolution, goal attainment rates, related health outcomes, and potential predictors.

## 2 Methods

This prospective, observational registry study was conducted from July 2020 to June 2024. Collaborating site enrollment occurred on a rolling basis. The primary outcome was breastfeeding duration, based on research conducted by Kair et al. (29). The protocol for this pilot study, based on prior ANDHII registry studies (19, 20), is detailed elsewhere. The present article presents results from the BFR.

The study was approved with exempt status by the University of North Florida (UNF) Institutional Review Board (IRB) (#1594452-2). Collaborating sites followed the approved protocol, and all BFR data in ANDHII were de-identified, per the Office for Human Research Protections Guidance on Research Involving Coded Private Information or Specimens Guidelines (30). An expert advisory group, composed of unpaid volunteers, supported study design and recruitment. Funding was provided through an Academy of Nutrition and Dietetics Foundation grant, awarded by the Women's Health and Pediatric Nutrition Dietetics Practice Groups. This article follows RECORD (31) guidelines for observational studies reporting routinely collected health data.

### 2.1 Collaborating sites and training

**Figure 1:**
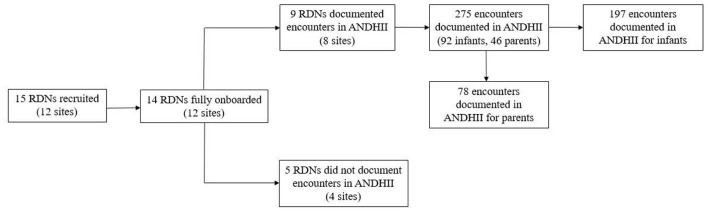
Flowchart of collaborating sites participation in the Breastfeeding Registry (BFR) Study from July 2020 to June 2024.

A convenient sample of twelve collaborating sites (14 RDNs) was included in the study (Figure 1); however, 4 sites (5 RDNs) withdrew due to time constraints, lack of response, or staff turnover. This analysis includes 92 infants from 8 sites (9 RDNs) in 6 U.S. states.

During orientation, RDNs completed a 5-h asynchronous training covering the study protocol, data collection procedures, the NCP and Terminology (NCP/T) (15), and ANDHII (17). The training served to establish baseline documentation skills. A score of 80% on a post-training knowledge assessment was required to earn continuing education credit and approval to commence data collection.

### 2.2 Inclusion and exclusion criteria

RDNs were required to: (1) hold state licensure to provide MNT, where applicable, (2) provide nutrition care to breastfeeding infants in their typical work environment, and (3) adhere to training and study protocols. Exclusion criteria included: (1) lack of RDN credentialing from the Commission on Dietetic Registration; (2) lack of required state licensure (where applicable); or (3) unwillingness/inability to document care in ANDHII.

### 2.3 Data collection and management

RDNs completed informed consent via Qualtrics and answered questions describing their education, clinical experience, training, and worksite characteristics. The survey generated a unique RDN code to link responses to ANDHII notes and enable matching of survey data on RDN and site characteristics to nutrition care outcomes. The code was included in the raw dataset and used only by the data analyst, who remained blinded to RDN identities. While providing care, RDNs remotely accessed ANDHII to document encounters. ANDHII guides NCPT documentation throughout the NCP steps (15). A selection of NCPT autopopulated in the nutrition assessment section to provide suggested documentation, but RDNs were encouraged to use any NCPT term they deemed appropriate. Autopopulated NCPT were determined by the expert advisory group. Each RDN documented at least one initial and one reassessment encounter for ten infants.

De-identified nutrition care data were exported from ANDHII as a raw data set and matched using system-generated codes. Raw data were used to describe breastfeeding duration and NCP outcomes, including NCPT documentation frequency, nutrition diagnosis improvement and resolution, nutrition goal attainment, impactful care plans (ICPs), and documentation quality. First presented by Long et al. (32) ICPs represent nutrition care patterns linked to the highest diagnosis improvement and resolution rates. Documentation quality was assessed using the validated Nutrition Care Process-Quality Evaluation and Standardization Tool (NCP-QUEST) (33), which scores 24 indicators (including the five NCP chain links) as 0 (not met) or 1 (met). Cumulative scores indicate documentation quality. Encounters with reassessments were rated as: Level A, High Quality (19-24 points), Level B, Medium Quality (13-18 points), and Level C, Low Quality (< 12 points).

### 2.4 Power calculation and sample size

Based on research by Kair et al. (29) a sample size of 60 infants was selected for feasibility of enrollment and 80% power (α = 0.05) for the primary outcome of breastfeeding duration. Clinical experience supported the feasibility of six RDNs documenting the care of at least ten infants.

### 2.5 Statistical analysis

Descriptive statistics are reported as frequencies (percentages) for categorical variables and means ± standard deviation for continuous and discrete variables; medians (IQR) were used for non-normally distributed data. These statistics were used to summarize the most frequently documented NCPT terms, quality of NCP documentation, nutrition diagnosis improvement and resolution, and goal attainment rates. Breastfeeding duration was defined as the total number of days between the documented NCPT *breastfeeding start (date)* and *breastfeeding stop (date)*. Diagnosis improvement and resolution rate was determined using diagnosis status labels documented at reassessment: *active-improving, active-unresolved, resolved*, and *discontinued*. NCPT terms for diagnoses with the highest improvement rates were used to identify ICPs. Goal attainment was assessed using documentation at reassessment: *met* or *unmet*.

Nonparametric tests were used to examine associations between sociodemographic, nutrition care, and NCP data. Mann-Whitney U tests were applied for continuous variables and chi-square tests were performed for categorical variables. Nutrition diagnosis improvement and resolution was coded binarily with *active-improving* or *resolved* diagnoses identified as “improved” and *active-not improved, unresolved*, and *discontinued* identified as “not improved.” Mixed-effects logistic regression was used to examine factors associated with nutrition diagnosis improvement and resolution. Planned analysis of the factors associated with nutrition goal attainment and breastfeeding duration were not possible due to insufficient documentation. Fixed effects, based on Colin et al. (19) and Lewis et al. (20) included NCP-QUEST score, diagnosis improvement, goal attainment, number of RDN visits, and presence of NCP chain link documentation. Random effects included RDN site type, education level, and lactation certification, as well as infant sex and birthweight.

For descriptive and chi-square analyses, missing data were included as “not documented” and retained for analysis to reflect clinical documentation patterns. For regression analyses, cases with missing data were excluded listwise. Records without a documented reassessment were excluded, as a lack of complete data results in the inability to estimate model parameters, reducing statistical power and introducing bias. Furthermore, this project did not aim to examine the lack of documentation. Analyses were conducted using IBM SPSS Statistics (Version 28.0.1.0) (34) and Stata 17MB (35). Statistical significance was defined as *p* < 0.05.

## 3 Results

### 3.1 Collaborating site and RDN characteristics

RDN and collaborating site characteristics are described in Supplementary Table 1. RDNs worked in community/public health clinics (78%; *n* = 7) or neonatal intensive care units (NICUs) (22%; *n* = 2). Most held a bachelor's degree (90%; *n* = 8) and a lactation credential (67%; *n* = 6). Among those with lactation credentials, the majority (67%; *n* = 4) reported having a CLC or CLE certification. The median (IQR) years of practice experience was 2 (0.5, 7) years and median (IQR) experience in the current position was 2 (0.5, 15 years). The median (IQR) number of RDNs employed at collaborating sites was 3 (1, 28), with 1 (0, 30) holding an advanced degree and 1 (0, 4) maintaining lactation credentials. Most (90%; *n* = 8) had not received training for ANDHII before the study, while the majority (78%; *n* = 7) had NCPT training. The NCPT was utilized at 67% of collaborating sites (*n* = 6).

### 3.2 Infant characteristics

The BFR dataset included nutrition care encounter data for 92 infants, of whom 71 had at least one documented reassessment encounter and nutrition diagnosis (Supplementary Figure 1). Most documented encounters were initial encounters (47%) and a first reassessment encounter (36%), however, RDNs documented up to seven reassessments for a single infant.

Infant sociodemographic and clinical characteristics are summarized in Table 1. Most infants were described as white (40%), male (41%), and non-Hispanic/Latino (46%). At baseline, the median age was 10 (6–20.5) days, increasing to 34.5 (27.5–60) days at reassessment. The average birth length was 46.3 ± 6.6 centimeters, and the average birthweight was 2,661.2 ± 1,030.6 grams. Infant weight showed a nonsignificant increase from initial (3,134.0 g ± 1,354.3 g) to reassessment (3,202.0 g ± 1,787.8 g) encounters (*p* = 0.203). A single NICU RDN was responsible for 25% of BFR data and over one-third of infants were hospitalized in the ICU.

**Table 1:** Characteristics of nutrition care process data documented by registered dietitian nutritionists (RDNs) in the Breastfeeding Registry Study and the relationship between documented NCP Data and nutrition diagnosis status.

**Variable**	**All Diagnoses (*N =* 69)**	**Diagnoses improved or resolved (*N =* 47)**	**Diagnoses not improved or resolved (*N =* 22)**	***P*-value**
**Sociodemographic data**				
Age, median (IQR)				
Baseline	(*N =* 66) 10 (6–20.5)	(*N =* 45) 10 (7–23)	(*N =* 21) 9 (2.5–13)	0.129
Reassessment	(*N =* 58) 34.5 (27.5–60)	*N =* 40 43.5 (28.5–73)	(*N =* 18) 30 (19.5–46)	0.023^*^
Sex, *N* (%)				0.004^*^
Male	28 (40.6)	13 (27.7)	15 (68.2)	
Female	22 (31.9)	17 (36.2)	5 (22.7)	
Not documented	19 (27.5)	17 (36.2)	2 (9.1)	
Race, *N* (%)				0.432
Asian	2 (2.9)	1 (2.1)	1 (4.5)	
Black	14 (20.3)	12 (25.5)	2 (9.1)	
White	40 (58)	24 (51.1)	16 (72.7)	
Not documented	13 (18.8)	10 (21.3)	3 (13.6)	
Ethnicity, *N* (%)				0.747
Hispanic/Latino	21 (30.4)	13 (27.7)	8 (36.4)	
Non-Hispanic/Latino	32 (46.4)	23 (48.9)	9 (40.9)	
Not Documented	16 (23.2)	11 (23.4)	5 (22.7)	
**Anthropometric and health data**				
Hospitalized in ICU, *N* (%)	25 (36.2)	17 (36.2)	8 (36.3)	0.988
Currently breastfeeding – yes, (includes exclusive, partial, or predominant), *N* (%)				
Baseline	61 (88.4)	45 (95.7)	16 (72.7)	0.021^*^
Reassessment	52 (75.4)	36 (76.6)	16 (72.7)	0.281
Body weight (grams), *x̄* ± SD				
Birthweight	(*N =* 58) 2,661.2 ± 1,030.6	(*N =* 39) 2,608.9 ± 1,027.6	(*N =* 19) 2,768.5 ± 1,056.4	< 0.001^*^
Baseline body weight	(*N =* 49) 3,035.8 ± 1,397.2	(*N =* 35) 3,116.7 ± 1,496.7	(*N =* 14) 2,833.6 ± 1,134.6	0.903
Reassessment body weight	(*N =* 40) 3,729.9 ± 2,203.5	(*N =* 26) 3,779.4 ± 1,761.9	(*N =* 14) 3,637.9 ± 2,929.5	0.292
Body length (centimeters), *x̄* ± SD				
Birth length	(*N =* 62) 46.3 ± 6.6	(*N =* 41) 46.1 ± 6.6	(*N =* 21) 46.8 ± 6.9	0.531
Baseline body length	(*N =* 38) 47.4 ± 7.3	(*N =* 25) 48.0 ± 7.1	(*N =* 13) 46.1 ± 7.9	0.564
Reassessment body length	(*N =* 37) 48.2 ± 8.6	(*N =* 24) 50.4 ± 8.6	(*N =* 13) 44.2 ± 7.4	0.053
**NCP documentation data**				
No. of visits by RDN within the study period, *x̄* ± SD	2.3 ± 0.8	2.4 ± 0.9	2.1 ± 0.5	0.279
Total care duration (minutes), *x̄* ± SD	73.7 ± 28.1	76.1 ± 29.6	69.2 ± 25.3	0.535
No. of nutrition problems documented, *x̄* ± SD	1.2 ± 0.4	1.2 ± 0.4	1.1 ± 0.4	0.351
Etiology documentation present, *N* (%)	59 (85.5)	38 (80.9)	21 (95.5)	0.108
Intervention documentation present, *N* (%)	58 (84.1)	38 (80.9)	20 (90.9)	0.288
Goal documentation present, *N* (%)	18 (26.1)	9 (19.1)	9 (40.9)	0.055
Goal status documentation present, *N* (%)	6 (33)	1 (11.1)	5 (55.6)	
Goal met/progress made toward goal	4 (66.7)	1 (100)	3 (60)	
Goal not met	2 (33.3)	0 (0)	2 (40)	
All 5 NCP chain links present, *N* (%)	7 (10.17)	6 (12.8)	1 (4.5)	0.554
Diagnosis-etiology link present, *N* (%)	50 (72.5)	35 (50.7)	15 (68.2)	0.586
Evidence-diagnosis link present, *N* (%)	26 (37.7)	22 (46.8)	4 (18.2)	0.022^*^
Intervention-goal link present, *N* (%)	20 (29)	11 (23.4)	9 (40.9)	0.135
Etiology-intervention link present, *N* (%)	52 (75.4)	37 (78.7)	14 (63.6)	0.184
Diagnosis-outcome link present, *N* (%)	69 (100)	47 (100)	22 (100)	–
NCP-QUEST audit score, x ± SD	13.4 ± 4.4	13.5 ± 4.6	13.1 ± 4.2	0.297
NCP-QUEST audit category, *N* (%)				0.017^*^
Low quality (≤12 points)	26 (37.7)	13 (27.7)	13 (59)	
Medium quality (13–18 points)	36 (52.2)	30 (63.8)	6 (27)	
High quality (19–24 points)	7 (10.1)	4 (8.5)	3 (13.6)	

IQR, interquartile range; ICU, intensive care unit; NCP, nutrition care process; NCP-QUEST, Nutrition Care Process Quality Evaluation and Standardization Tool. The “*” refers to p-values of < 0.05 which are considered significant.

### 3.3 Breastfeeding duration

Breastfeeding (partial, predominant, exclusive), daily breastmilk feeding attempts, use of donor milk, and breastfeeding start/stop dates were inconsistently documented. Start/stop breastfeeding NCPT was documented for 10 (14%) infants. For these 10 infants, the average breastfeeding duration was 34.2 ± 7.5 days, slightly exceeding the average care duration in the BFR, 25.8 ± 32.2 days. Although the duration of breastfeeding was the primary outcome of this project, limited documentation by RDNs prevented further exploration of this variable. Most infants were breastfeeding at baseline (88%) and reassessment (75%) (Table 1). More infants with improved nutrition diagnoses were breastfeeding at the initial encounter (96%) compared to those without diagnosis improvement (73%; *p* = 0.021). Donor milk intake increased between initial (15%) and reassessment (23%) encounters.

### 3.4 NCP outcomes

Over 3,000 NCPT terms were documented by RDNs. The five most frequently documented terms for each NCP step are detailed in Supplementary Table 2. Excluding demographic and anthropometric data, the most frequently documented nutrition assessment terms were *mother's expressed breastmilk intake* (8%) and *exclusive breastfeeding* (6.5%). *Breastfeeding difficulty* was the most frequent problem (31% of problem terms) and etiology (18% of etiology terms). The most frequently documented signs and symptoms term was *energy estimated intake from oral nutrition in 24 h* (17% of signs and symptoms terms). The most frequent nutrition intervention was *modify breastfeeding attempts* (14% of nutrition intervention terms). The leading monitoring and evaluation term was *breastmilk feeding attempts in 24 h* (14% of monitoring and evaluation terms).

**Figure 2:**
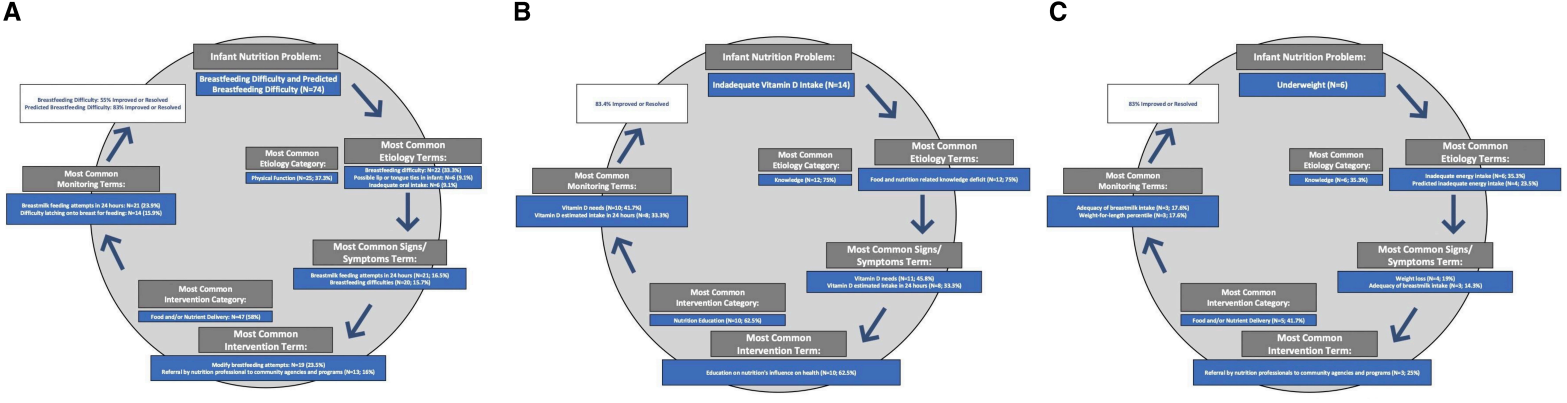
Impactful Care Plans for the nutrition diagnoses with the highest rate of resolution in the Breastfeeding Registry Study: Breastfeeding Difficulty/Predicted Breastfeeding Difficulty **(A);** Underweight **(B**); Inadequate Vitamin D Intake **(C)**.

Approximately 68% of nutrition diagnoses were *active-improving* or *resolved* at reassessment. Infants without improvement in nutrition diagnosis were significantly younger (31.3 days) compared to those with improved nutrition diagnoses (56.7 days; *p* = 0.023) (Table 1). The group with unimproved nutrition diagnoses had a significantly higher proportion of male infants (68.2% vs. 27.7%, *p* = 0.004) and lower average birthweight (2768.5 g vs. 3308.9 g, *p* < 0.001). The most frequently improved or resolved diagnoses were *breastfeeding difficulty* (54.6%), *predicted breastfeeding difficulty* (83.4%), *inadequate vitamin D intake* (83.4%), and *underweight* (83.4%). ICPs were identified to illustrate complete NCP cycles and the respective NCPT that lead to measurable diagnosis improvement (Figure 2). Encounters with *breastfeeding difficulty* and *predicted breastfeeding difficulty* were combined in a single ICP due to documentation similarities. Approximately 26% of encounters included documentation of nutrition goals; of these, 33% included goal status at reassessment, with 67% indicating progress toward the goal (Table 1).

Among infants with at least one reassessment (*n* = 69), the average NCP-QUEST score was 13.4 (Level B, medium quality) (Table 1). No significant differences in audit scores were observed between encounters with improved vs. unimproved diagnoses; however, NCP-QUEST rating categories differed significantly by diagnosis status (*p* = 0.017). Supplementary Figure 2 details the distribution of the 24 NCP-QUEST indicators. Diagnosis-outcomes link documentation was present in all encounters. Most included diagnosis-etiology (73%) and etiology-intervention (75%) links, while documentation of evidence-diagnosis (37%) and intervention-goal (29%) links were limited. Evidence-diagnosis link documentation was significantly more common for improved or resolved diagnoses (*p* = 0.022). Only 7% of encounters included all five NCP chain links.

A mixed-effects logistic regression model examined factors associated with nutrition diagnosis improvement and resolution (Table 2). The first nutrition diagnosis recorded for infants with a reassessment encounter was included, resulting in 60 encounters documented by 9 RDNs. The intervention-goal link and breastfeeding duration were excluded due to insufficient data, and the diagnosis-outcome link was excluded due to lack of variability. Each 1-point increase in the NCP-QUEST score was associated with a 58% increase in the odds of diagnosis improvement (OR = 1.57, 95% CI [1.02, 2.45], *p* = 0.042). Each additional RDN visit was associated with a 77% increase in the odds of diagnosis improvement (OR = 1.77, 95% CI [0.34, 9.33, *p* = 0.049). A master's degree was associated with lower odds of diagnosis improvement (OR = 0.004, 95% CI [0.00, 0.54], *p* = 0.03), although a single RDN within the BFR held a master's degree. Higher birthweight was also linked to reduced odds of diagnosis resolution (OR = 0.998, 95% CI [0.0996–0.9997], *p* = 0.024. While statistically significant, these associations should be interpreted with caution, as they likely reflect model instability, driven by the small sample size, and differences in infant acuity. Model fit was modest (AIC = 55.34, BIC = 72.8) with a non-significant Wald chi-square test [$X_{\left(7\right)}^{2}$ = 9.67, *p* = 0.3782] suggesting other unexamined variables may influence nutrition diagnosis improvement and resolution.

**Table 2:** Factors associated with nutrition diagnosis improvement in the breastfeeding registry study.

**Variable**	**OR**	**SE**	***Z***	***P***	**95% CI**	
					**Upper**	**Lower**
NCP-QUEST score	1.577	0.35	2.03	0.042^*^	1.016	2.446
Number of visits	1.771	1.50	0.67	0.049^*^	0.336	9.327
Diagnosis-etiology link	6.703	8.85	1.44	0.149	0.505	88.998
Evidence-diagnosis link	0.198	0.28	−1.13	0.26	0.012	3.327
Intervention-goal link	0.148	0.25	−1.15	0.251	0.006	3.875
RDN education level	0.004	0.01	−2.21	0.03^*^	0.000	0.539
Lactation certification	2.139	2.84	0.57	0.57	0.158	28.869
Infant sex	0.164	0.16	−1.81	0.07	0.023	1.162
Birthweight	0.998	0.00	−2.26	0.02^*^	0.996	0.999

OR, Odds Ratio; SE, Standard Error; CI, Confidence Interval; NCP-QUEST, Nutrition Care Process Quality Evaluation and Standardization Tool; RDN, registered dietitian nutritionist. The “*” refers to p-values of < 0.05 which are considered significant.

## 4 Discussion

The aims of this study were to: (1) identify the most frequently documented NCPT; (2) evaluate the quality of NCP documentation; and (3) examine nutrition diagnosis improvement and resolution, goal attainment rates, related health outcomes, and potential predictors. Findings indicated that the care provided to breastfeeding infants by RDNs in the BFR effectively improved infants' nutrition diagnoses. Greater odds of nutrition diagnosis improvement and resolution were observed with increasing NCP-QUEST scores and additional RDN visits. The nutrition problems with the highest rate of improvement and resolution were *breastfeeding/predicted breastfeeding difficulty, underweight*, and *inadequate vitamin D intake*. These prevalent nutrition diagnoses align with common barriers to the initiation and continuation of breastfeeding reported in the literature (11–13).

### 4.1 Breastfeeding outcomes

The primary outcome was breastfeeding duration; however, limited documentation of breastfeeding data in the BFR hindered the planned full examination, its associated factors, and RDNs' impact on breastfeeding outcomes. Establishing standardized variables for documenting infant feeding practices aligned with WHO/UNICEF (5) standards, such as initiation date, exclusivity, and cessation date, would improve data tracking and support exclusive breastfeeding promotion efforts within pediatric primary care (36).

A 2021 study reported that a sample of U.S. infants were breastfed for an average of 6.9 months (37). Observed breastfeeding duration in the BFR falls short of these findings and global breastfeeding standards, exclusive breastfeeding for 6 months with continued breastfeeding through two years and beyond (5). Shorter exclusive breastfeeding duration carries important public health implications, including increased risk of infant illness and chronic disease among infants and parents (1, 5, 38). To minimize RDN burden, the BFR study required documentation of only an initial visit and one follow-up, which may have limited the completeness of breastfeeding data documentation. Future studies should therefore expand follow-up documentation requirements and lengthen study duration to strengthen findings.

Most infants (88%; *n* = 61) were breastfeeding in some capacity at initial and reassessment encounters, on par with the U.S. average of infants who are ever breastfed (Table 1) (39). Breastfeeding rates at the initial assessment were significantly higher among infants with improved nutrition diagnoses (96%; *n* = 45) compared to infants without diagnosis improvement and resolution (73%; *n* = 16; *p* = 0.021), suggesting that early breastfeeding may have contributed to better nutrition outcomes in the BFR. This aligns with existing literature on the health benefits of early human milk feeding (1, 5, 38). Although donor milk intake increased between initial and reassessment encounters, it remains unclear whether BFR RDNs played a direct role in recommending or facilitating access to donor milk. To draw such conclusions, more detailed documentation of nutrition care, such as nutrition interventions and goals related to the use of donor milk, is necessary. While there is limited evidence regarding the number of U.S. infants utilizing donor milk, an increase in donor human milk use in clinical settings has been reported within the last decade (40, 41). Current WHO/UNICEF recommendations for infant feeding place direct breastfeeding as the first priority, followed by feeding with the mother's expressed breastmilk. When these options are not possible, donor milk, wet nursing, or relactation may be considered, based on the family's preferences, cultural norms, and the safety of providing human milk in the context of transmissible conditions, such as human immunodeficiency virus (HIV) and coronavirus disease (COVID). Importantly, WHO/UNICEF advise that all these options be explored before turning to human milk substitutes (infant formula) (5, 42).

### 4.2 Predictors of diagnosis improvement and resolution

Infants whose nutrition diagnoses did not improve had significantly lower average birthweight and were more often male (Table 1). The association with sex likely reflects the small sample size, while the proportion of low birthweight infants with unimproved diagnoses aligns with known complications of low birthweight and may indicate higher acuity of care (43). Although infant sex was not a predictor of diagnosis resolution, higher birthweight was unexpectedly associated with lower odds of diagnosis resolution (OR = 0.998, 95% CI [0.0996–0.9997], *p* = 0.024). This counterintuitive finding is likely influenced by differences in acuity, study requirements of only one initial and one follow-up assessment, and the limited sample size.

Similar to prior research (19, 20), higher quality NCP documentation predicted better outcomes: each one-point increase in NCP-QUEST score was associated with a 58% greater likelihood of diagnosis improvement and resolution (OR = 1.58, 95% CI [1.01, 2.45], *p* = 0.042) (Table 2) (19, 20). Consistent with findings by Lewis et al. (20) each additional nutrition visit was associated with a 77% greater likelihood of diagnosis improvement (OR = 1.77, 95% CI [0.34, 9.33], *p* = 0.049), suggesting that more frequent contact with RDNs improved health-related outcomes for infants in the BFR (Table 2). Notably wide confidence intervals among predictors are likely due to the small sample size of this pilot registry study and may limit interpretation of the statistical model.

Previous research has emphasized the critical role of RDNs in supporting breastfeeding (9, 10, 44–47), though there is an opportunity to improve knowledge and training in this practice area (48–50). BFR findings further highlight the role of the RDNs in enhancing public health outcomes through nutrition care and lactation support.

### 4.3 Impactful care plans

Nearly 70% of nutrition diagnoses in the BFR were improved at reassessment (Table 1), an improvement rate that exceeds those previously reported in other dietetics registry studies (19, 20). The BFR study presents the first known specific description of effective nutrition care patterns delivered to breastfeeding infants. Impactful care plans (Figure 2) can be utilized as a practice tool by lactation RDNs. In most cases, *breastfeeding* and *predicted breastfeeding difficulty* diagnoses improved or resolved. Notably, RDNs most frequently identified physical-function as root causes for breastfeeding difficulties, which are consistent with common breastfeeding difficulties reported in the literature: cracked or sore nipples, latching problems, insufficient milk supply, and breast engorgement (11–13). BFR RDNs provided support through modification of latch and breastmilk feeding attempts as part of the nutrition intervention.

In this study, RDN care improved *underweight* and *inadequate vitamin D intake* nutrition diagnoses, both of which carry significant implications for infant and long-term health. Underweight in infancy is linked to higher nutrition risk throughout childhood (51), while inadequate vitamin D intake can result in deficiency or rickets, compromising bone health and development (52). These nutrition problems represent major public health challenges, as their impact extends beyond infancy and contributes to greater healthcare burden (53, 54), yet BFR findings demonstrate that RDNs frequently improved *underweight* and *inadequate vitamin D intake* through tailored nutrition education. The effectiveness of RDNs in addressing *breastfeeding difficulties, underweight*, and *inadequate vitamin D intake* highlights their essential role in promoting breastfeeding, infant health, and public health. To our knowledge, this is the first study to examine NCP documentation patterns in lactation nutrition, addressing a critical evidence gap regarding how RDNs support breastfeeding infants.

### 4.4 NCP documentation quality

Despite pre-study training, documentation quality in the BFR was suboptimal, with approximately 90% (*n* = 62) of documented encounters categorized as low- and medium-quality according to the NCP-QUEST (Table 1). Nutrition etiologies (85%; *n* = 59) and interventions (84%; *n* = 58) were well-documented, however, nutrition goals (26%; *n* = 18) were not. Only 10% (*n* = 7) of encounters in the BFR contained complete NCP chains, in contrast to previous registry studies reporting approximately 50% of encounters as complete NCP chains (19, 20). Despite the opportunity for improving NCP documentation quality in the BFR, the problem resolution rate in the BFR was higher than rates previously reported and each one-point increase in NCP-QUEST score was associated with a 58% increase in the odds of diagnosis improvement and resolution.

### 4.5 Limitations

As a U.S.-based pilot registry study relying on a convenience sample, findings may not be generalizable to the global population, and the high withdrawal rate may have introduced selection bias. All data came from ANDHII chart reviews, which may not capture the full course of care; documentation was limited to standardized NCPT entries, and RDNs may have documented only partially due to time constraints (18) or the two-encounter study requirement. Analysis of breastfeeding outcomes was further constrained by insufficient documentation of key data. Despite rigorous modeling, some model instability was observed, including the counterintuitive associations between a master's degree and lower odds of nutrition diagnosis improvement, as well as higher birthweight and less improvement. The association with education likely reflects a single RDN with higher-acuity patients whose problems were less likely to improve within the study timeframe. Similarly, the birthweight finding may be influenced by differences in patient acuity and the limited sample size. Wide confidence intervals further reflect the limited sample.

Despite these limitations, standardized NCPT data from multiple RDNs and practice sites enhances clinical relevance and offers essential insight into real-world lactation nutrition care. While not generalizable, these findings are hypothesis-generating and lay the groundwork for future practice-based research in this underexplored area of dietetics.

### 4.6 Implications for practice

Our findings add to evidence that higher-quality NCP documentation supports high-quality, targeted nutrition care, reinforcing the need to adopt the NCPT in lactation and broader dietetics practice. To effectively track exclusive breastfeeding duration, we recommend documentation of a core outcomes set (COS): breastfeeding initiation date, extent (exclusive, predominant, or partial), route(s) of human milk administration (i.e., breast, bottle, or enteral feedings), and cessation date. COS are sets of data elements designed to assess outcomes for patients with the same health interest or condition, allowing researchers to report the same outcomes across studies and draw stronger conclusions about the effectiveness of interventions for a specialty area (55). We urge adoption of this COS in the routine documentation of infant nutrition care to enhance data quality and enable standardized tracking of breastfeeding practices across dietetics, pediatrics, and public health research. This standardization will strengthen the alignment of individual nutrition care to broader public health goals and outcomes. The Core Outcome Measures in Effectiveness Trials (COMET) Initiative (56), which houses a database of COS-related studies and completes regular updates to a systematic review of all related research, reports no COS have been developed for breastfeeding research. Thus, there is a need for further research in this area and the proposed COS for breastfeeding research is consistent with the guidance of the COMET Initiative (56), which emphasizes stakeholder involvement in outcome identification and consensus-building to enhance comparability across studies.

Continued development of ICPs is warranted to clarify their utility in research and practice. ICPs are likely a tool for RDNs to apply to patient care while also promoting greater involvement in practice-based research. Still, there is a need to clearly define the improvement ICPs showcase, in terms of both statistical and clinical significance. With no evidence-based nutrition practice guidelines or Academy Position papers on lactation nutrition, BFR findings may inform continuing education and training standards to support practice improvement and strengthen RDNs' impact on public health.

## 5 Conclusion

These findings highlight the critical role of RDNs in supporting breastfeeding and advancing public health. In applying the NCP and utilizing standard terminology, RDNs addressed common barriers to meeting breastfeeding goals, achieving an 80% diagnosis improvement rate in the BFR. These findings offer an initial foundation for future research into how lactation nutrition care may advance both clinical and public health outcomes. Identified ICPs for *breastfeeding difficulty, underweight*, and *inadequate vitamin D intake* serve as practical resources for RDNs. This study is first known effort to examine NCP documentation patterns in lactation nutrition, providing novel evidence of how RDNs address breastfeeding challenges and highlighting the importance of robust documentation and practice-based research in alignment with global breastfeeding standards. Continued research, using standardized breastfeeding data, longer-duration follow-up, and larger samples is essential to strengthen the evidence base, inform dietetics training and practice, and optimize RDNs' impact on breastfeeding outcomes and public health.

## Data Availability

The raw data supporting the conclusions of this article will be made available by the authors, without undue reservation.
