# Supplementary material for: Higher quality nutrition care process documentation predicts nutrition diagnosis improvement in the Academy of Nutrition and Dietetics breastfeeding registry study
Source: Front Nutr. 2025 Nov 17;12:1632931. doi: 10.3389/fnut.2025.1632931 (PMC12667241; doi:10.3389/fnut.2025.1632931)
Supplement: Supplementary file 1 [file Supplementary_file_1.docx]

Supplementary Material


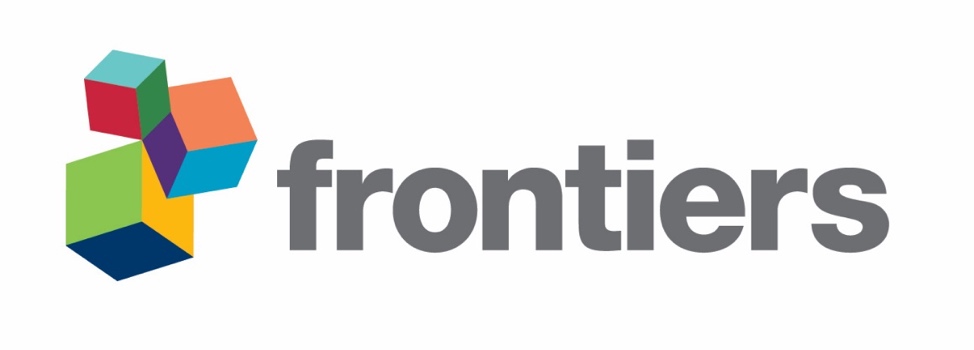


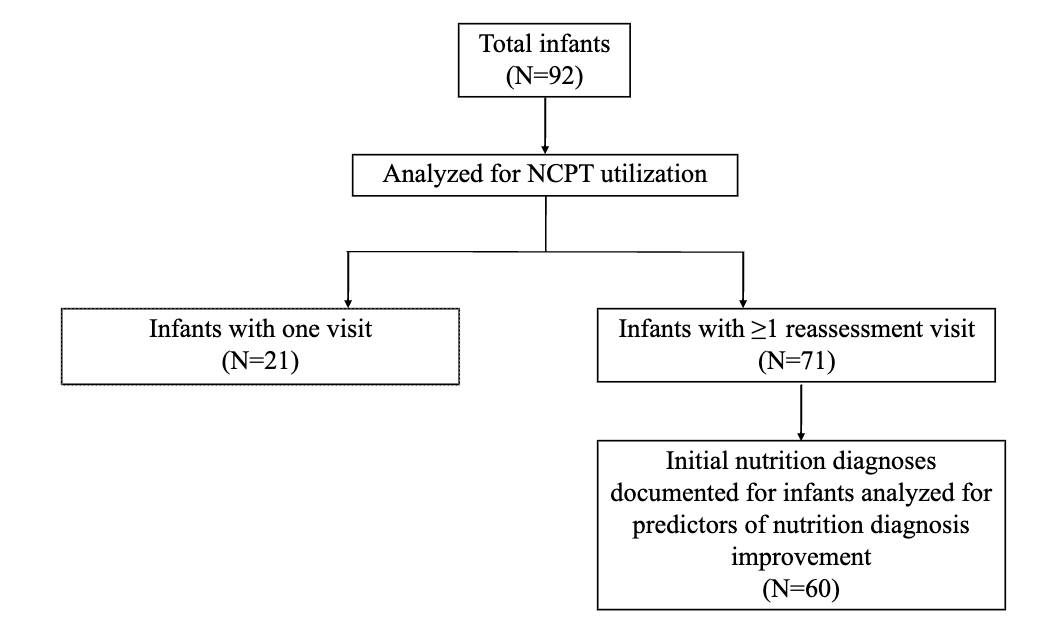

**Supplementary Figure 1.** Flow chart illustrating the process of analyzing nutrition care data documented in the Academy of Nutrition and Dietetics Health Informatics Infrastructure for infants in the Breastfeeding Registry Study (N=92). NCPT, Nutrition Care Process Terminology^9^

**Supplementary Table 1.** Registered Dietitian Nutritionist (RDN) and Collaborating Site Characteristics for the Breastfeeding Registry (BFR) Study.

|  | **N (%)** | | |
| --- | --- | --- | --- |
| **RDN Race** | | | |
| White or Caucasian | 9 (100) | | |
| **RDN Education Level** | | | |
| Bachelor’s Degree | 8 (89) | | |
| Master’s Degree | 1 (11) | | |
| **RDN Lactation Certification - Yes** | 6 (67) | | |
| CLC or CLE | 4 (44) | | |
| IBCLC | 2 (22) | | |
| **Facility Type** |  | | |
| Community/Public Health | 7 (78) | | |
| Neonatal Intensive Care Unit | 2 (22) | | |
| **Prior ANDHII Training – Yes** | 1 (11) | | |
| **Prior NCP/T Training - Yes** | 7 (78) | | |
| **NCP/T Utilized at Collaborating Site – Yes** | 6 (67) | | |
|  | **Median (IQR)** | **Minimum** | **Maximum** |
| **Years of RDN experience** | 2 (6) | 0.5 | 15 |
| **Years working at collaborating site** | 2 (5) | 0.5 | 15 |
| **Years working in current position** | 2 (6) | 0.5 | 15 |
| **Number of RDNs employed at collaborating site** | 3 (10) | 1 | 28 |
| **Number of RDNs with advanced degrees employed at collaborating site** | 1 (3) | 0 | 30 |
| **Number of RDNs with lactation credentials at collaborating site** | 1 (3) | 0 | 4 |

_________________________________________________________________________________

Abbreviations: CLC, Certified Lactation Counselor. CLE, Certified Lactation Educator. IBCLC, International Board-Certified Lactation Consultant. ANDHII, Academy of Nutrition and Dietetics Health Informatics Infrastructure. NCP/T, Nutrition Care Process and Terminology.


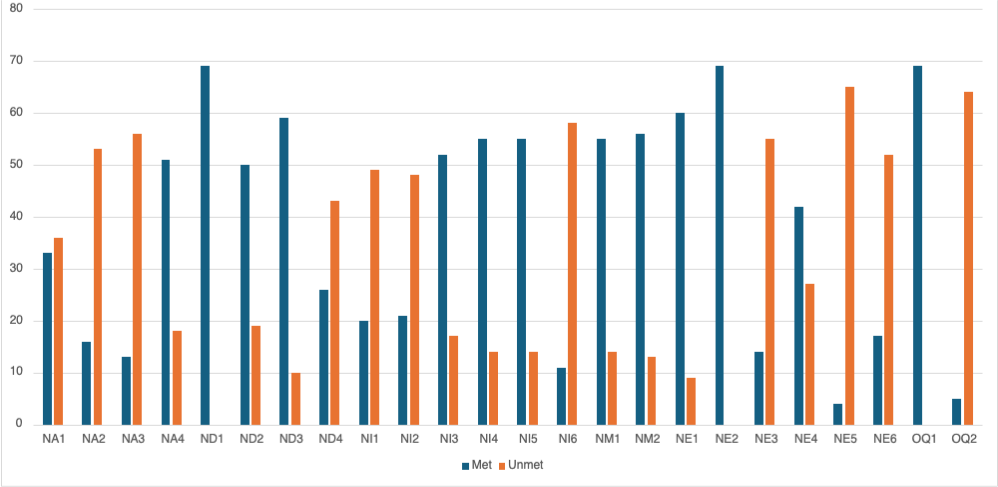

**Supplementary Figure 2.** Distribution of Nutrition Care Process Quality Evaluation and Standardization Tool (NCP-QUEST)^26^ indicators from the audit of nutrition care documentation (N=69) in the Breastfeeding Registry (BFR). All encounters in the BFR were documented within the Academy of Nutrition and Dietetics Health Informatics Infrastructure (ANDHII).^8^ This figure includes matched initial and reassessment encounters for infants with at least one documented nutrition diagnosis. Specific indicators correspond to the five NCP chain links: ND2 (evidence-diagnosis link), ND4 (diagnosis-etiology link), NI1 (etiology-intervention link), NI3 (intervention-goal link), and NE2 (diagnosis-outcomes link). NA= nutrition assessment; ND= nutrition diagnosis; NI= nutrition intervention; NM= nutrition monitoring; NE= nutrition evaluation; OQ= overall quality.

**Supplementary Table 2.** Distribution of the top five most frequently documented Nutrition Care Process Terminology (NCPT)^9^ for infants (N=92) in the Breastfeeding Registry (BFR) Study.

|  | **Domain/ Category** | **NCPT Term** | **N (%)** |
| --- | --- | --- | --- |
| **Nutrition Assessment** | | | |
| Assessment NCPT  (N=2,229 terms) | Client History | Age | 184 (8.2) |
|  | Food or Nutrition-Related History | Mothers expressed breastmilk intake | 179 (8.0) |
|  | Client History | Gender | 163 (7.3) |
|  | Food or Nutrition-Related History | Exclusive breastfeeding | 145 (6.5) |
|  | Anthropometric Measurements | Weight | 138 (6.2) |
|  | Other NCPT documented | | 1420 (63.7) |
| **Nutrition Diagnosis** | | | |
| Problem NCPT  (N=180 terms) | Clinical | Breastfeeding difficulty | 56 (31.1) |
|  | Intake | Inadequate oral intake | 29 (16.1) |
|  | Clinical | Predicted breastfeeding difficulty | 18 (10) |
|  | Intake | Inadequate energy intake | 15 (8.3) |
|  | Intake | Inadequate vitamin D intake | 14 (7.8) |
|  | Other NCPT documented | | 48 (26.7) |
| Etiology NCPT*  (N=160 terms) | Physical-Function | Breastfeeding difficulty | 28 (17.5) |
|  | Knowledge | Food and nutrition related knowledge deficit | 25 (15.6) |
|  | Behavior | Disordered eating pattern | 14 (8.8) |
|  | Physiologic-Metabolic | Inadequate energy intake | 14 (8.8) |
|  | Physiologic-Metabolic | Increased nutrient needs | 13 (7.2) |
|  | Physical-Function | Predicted breastfeeding difficulty | 13 (7.2) |
|  | Other NCPT documented | | 53 (33.1) |
| Signs/Symptoms NCPT  (N=292 terms) | Food or Nutrition-Related History | Energy estimated intake from oral nutrition in 24 hours | 50 (17.1) |
|  | Food or Nutrition-Related History | Breastmilk feeding attempts in 24 hours | 35 (12) |
|  | Food or Nutrition-Related History | Adequacy of infant formula intake | 22 (7.5) |
|  | Food or Nutrition-Related History | Breastfeeding difficulties | 19 (6.5) |
|  | Food or Nutrition-Related History | Difficulty latching onto breast for feeding | 15 (5.1) |
|  | Other NCPT documented | | 151 (51.7) |
| **Nutrition Intervention** | | | |
| Intervention NCPT  (N=237 terms) | Food and/or Nutrient Delivery | Modify breastfeeding attempts | 32 (13.5) |
|  | Coordination of Nutrition Care | Collaboration by nutrition professional with other providers | 22 (9.3) |
|  | Coordination of Nutrition Care | Referral by nutrition professional to community agencies and programs | 21 (8.9) |
|  | Nutrition Education | Education on nutrition's influence on health | 19 (8) |
|  | Food and/or Nutrient Delivery | Evaluation of breastfeeding | 14 (5.9) |
|  | Other NCPT documented | | 129 (54.4) |
| **Nutrition Monitoring and Evaluation** | | | |
| Reassessment NCPT  (N=227 terms) | Food or Nutrition-Related History | Breastmilk feeding attempts in 24 hours | 31 (13.7) |
|  | Food or Nutrition-Related History | Difficulty latching onto breast for feeding | 19 (8.4) |
|  | Food or Nutrition-Related History | Energy estimated intake from oral nutrition in 24 hours | 15 (6.6) |
|  | Food or Nutrition-Related History | Inadequate flow of breastmilk | 14 (6.2) |
|  | Food or Nutrition-Related History | Breastfeeding difficulties | 10 (4.4) |
|  | Food or Nutrition-Related History | Infant formula intake | 10 (4.4) |
|  | Comparative Standards | Vitamin D needs | 10 (4.4) |
|  | Other NCPT documented | | 118 (52.0) |

Nutrition care encounter documentation was analyzed from the Academy of Nutrition and Dietetics Health Informatics Infrastructure (ANDHII).^8^ This table reflects NCPT from a total of 197 initial and reassessment encounters, with RDNs documenting multiple terms across Nutrition Care Process^9^ steps. The five most frequently documented NCPT terms are listed; in case of a tie, terms appear in alphabetical order. *There are no nutrition etiology NCPT terms. Nutrition etiologies were free texted in ANDHII.
